# Inhibition of Plasminogen Activator Inhibitor-1 Activation Suppresses High Fat Diet-Induced Weight Gain *via* Alleviation of Hypothalamic Leptin Resistance

**DOI:** 10.3389/fphar.2020.00943

**Published:** 2020-06-24

**Authors:** Shinichiro Hosaka, Tetsuya Yamada, Kei Takahashi, Takashi Dan, Keizo Kaneko, Shinjiro Kodama, Yoichiro Asai, Yuichiro Munakata, Akira Endo, Hiroto Sugawara, Yohei Kawana, Junpei Yamamoto, Tomohito Izumi, Shojiro Sawada, Junta Imai, Toshio Miyata, Hideki Katagiri

**Affiliations:** ^1^Department of Metabolism and Diabetes, Tohoku University Graduate School of Medicine, Sendai, Japan; ^2^Department of Molecular Medicine and Therapy, United Center for Advanced Research and Translational Medicine, Tohoku University Graduate School of Medicine, Sendai, Japan

**Keywords:** plasminogen activator inhibitor-1, obesity, leptin resistance, arcuate nucleus, food intake, thermogenesis

## Abstract

Leptin resistance is an important mechanism underlying the development and maintenance of obesity and is thus regarded as a promising target of obesity treatment. Plasminogen activator inhibitor 1 (PAI-1), a physiological inhibitor of tissue-type and urokinase-type plasminogen activators, is produced at high levels in adipose tissue, especially in states of obesity, and is considered to primarily be involved in thrombosis. PAI-1 may also have roles in inter-organ tissue communications regulating body weight, because PAI-1 knockout mice reportedly exhibit resistance to high fat diet (HFD)-induced obesity. However, the role of PAI-1 in body weight regulation and the underlying mechanisms have not been fully elucidated. We herein studied how PAI-1 affects systemic energy metabolism. We examined body weight and food intake of PAI-1 knockout mice fed normal chow or HFD. We also examined the effects of pharmacological inhibition of PAI-1 activity by a small molecular weight compound, TM5441, on body weight, leptin sensitivities, and expressions of thermogenesis-related genes in brown adipose tissue (BAT) of HFD-fed wild type (WT) mice. Neither body weight gain nor food intake was reduced in PAI-1 KO mice under chow fed conditions. On the other hand, under HFD feeding conditions, food intake was decreased in PAI-1 KO as compared with WT mice (HFD-WT mice 3.98 ± 0.08 g/day *vs* HFD-KO mice 3.73 ± 0.07 g/day, *P* = 0.021), leading to an eventual significant suppression of weight gain (HFD-WT mice 40.3 ± 1.68 g *vs* HFD-KO mice 34.6 ± 1.84 g, *P* = 0.039). Additionally, TM5441 treatment of WT mice pre-fed the HFD resulted in a marked suppression of body weight gain in a PAI-1-dependent manner (HFD-WT-Control mice 37.6 ± 1.07 g *vs* HFD-WT-TM5441 mice 33.8 ± 0.97 g, *P* = 0.017). TM5441 treatment alleviated HFD-induced systemic and hypothalamic leptin resistance, before suppression of weight gain was evident. Moreover, improved leptin sensitivity in response to TM5441 treatment was accompanied by increased expressions of thermogenesis-related genes such as *uncoupling protein 1* in BAT (HFD-WT-Control mice 1.00 ± 0.07 *vs* HFD-WT-TM5441 mice 1.32 ± 0.05, *P* = 0.002). These results suggest that PAI-1 plays a causative role in body weight gain under HFD-fed conditions by inducing hypothalamic leptin resistance. Furthermore, they indicate that pharmacological inhibition of PAI-1 activity is a potential strategy for alleviating diet-induced leptin resistance in obese subjects.

## Introduction

An alarming increase in the number of obese subjects has become a major public health concern worldwide in recent decades, since obesity increases cardiovascular risks with increased prevalence of diabetes, hyperlipidemia, and hypertension, collectively termed the metabolic syndrome. Two key components determining body weight are energy intake and energy expenditure (Yamada et al., 2008). These components are regulated by inter-organ/tissue communications (Katagiri et al., 2007). Adipokines constitute a group of cytokines that are synthesized predominantly in adipose tissues and participate in such metabolic communications (Abella et al., 2017). Among these factors, leptin is produced in proportion to body fat mass and is released into the bloodstream. Increased circulating leptin typically reduces feeding and promotes energy expenditure by targeting hypothalamic nuclei that are important for energy homeostasis (Cui et al., 2017). Obesity, however, is associated with leptin resistance, a state in which elevated circulating leptin and exogenously delivered leptin are both less effective in creating satiety and suppressing food intake (Liu et al., 2015). Thus, on the basis of the pathophysiology underlying obesity, alleviation of leptin resistance is a reasonable and long-awaited therapeutic strategy for treating obesity, the anticipated outcome of which would be normalization of energy metabolism regulation. Despite long-standing research efforts, however, such pharmacologic agents have yet to be developed.

Plasminogen activator inhibitor 1 (PAI-1) was primarily identified as a physiological inhibitor of tissue-type and urokinase-type plasminogen activators, though it may also have roles in inter-organ/tissue communications as an adipokine. PAI-1 is produced in adipose tissue (Khandekar et al., 2011) and its levels are elevated in both adipose tissues and the plasma of obese mice and humans (Bodary, 2007). Intraperitoneal administration of PAI-1 was reported to increase food intake during refeeding after a 24-h fast (Kenny et al., 2013). In addition, transgenic mice overexpressing PAI-1 in gastric parietal cells, which results in plasma PAI-1 elevation, reportedly show both hyperphagia and obesity (Kenny et al., 2013). In high fat diet (HFD)-fed PAI-1 knockout (KO) mice, reduced weight gain, decreased plasma leptin levels, and increased energy expenditure (when divided by body weight) were observed (Ma et al., 2004). Therefore, PAI-1 is suggested to participate in body weight regulation by modulating leptin effects such as energy intake and/or energy expenditure, but the pathophysiological mechanisms whereby PAI-1 modulates leptin sensitivities and energy metabolism have not as yet been elucidated.

TM5441 is a low molecular weight compound which was previously confirmed to specifically and dose-dependently inhibit PAI-1 (Boe et al., 2013). The known phenotypic effects of TM5441 *in vivo* include protection against hypertension and vascular senescence in Nω-nitro-l-arginine methyl ester (L-NAME)-induced hypertensive mice (Boe et al., 2013), prolongation of lifespan in klotho null mice (Eren et al., 2014), and improved renal function and morphology in streptozotocin-induced diabetic mice (Jeong et al., 2016). In addition, a recent study demonstrated that TM5441 treatment exerted a suppressive effect on body weight gain, when provided together with HFD-feeding (Piao et al., 2016). Thus, PAI-1 inhibition by TM5441 treatment is a potential preventive/therapeutic strategy for obesity. However, the molecular mechanisms underlying the actions of PAI-1 inhibitors in suppressing body weight gain remain essentially unexplored.

In the present study, we, therefore, attempted to elucidate how PAI-1 affects systemic energy metabolism employing both PAI-1 KO mice and PAI-1 inhibitor-treated mice. We identified a novel pathophysiological role of PAI-1 in aggravating leptin resistance and demonstrated a novel therapeutic PAI-1 inhibitor action exerted by alleviating HFD-induced leptin resistance.

## Materials and Methods

### Animals

C57BL/6J mice were purchased from Japan SLC (Shizuoka, Japan). PAI-1 KO mice (background strain C57BL/6J; strain name B6.129S2-Serpine1tm1Mlg/J; stock no. 002507) were obtained from Jackson Laboratory (Bar Harbor, ME, USA). Mice were housed under controlled temperature and humidity conditions on a 12-h light, 12-h dark cycle. The animals were housed individually and given standard laboratory diet (65% carbohydrate, 4% fat, 24% protein) and water *ad libitum* unless otherwise noted. All animal studies were conducted in accordance with the institutional guidelines for animal experiments at Tohoku University.

### High Fat Diet

Two types of HFD were used in this study. A safflower oil-rich HFD (Yamada et al., 2006) (32% safflower oil, 33.1% casein, 17.6% sucrose, and 5.6% cellulose) was purchased from Oriental Yeast (Tokyo, Japan) and a lard-rich D12492 HFD (31.7% lard, 25.8% casein, 8.9% sucrose, and 6.5% cellulose) was purchased from Research Diet (New Brunswick, NJ, USA). Both of these HFD were purchased in powder form and then shaped into pellets in our laboratory.

### Preparation of TM5441

**Figure 1:**
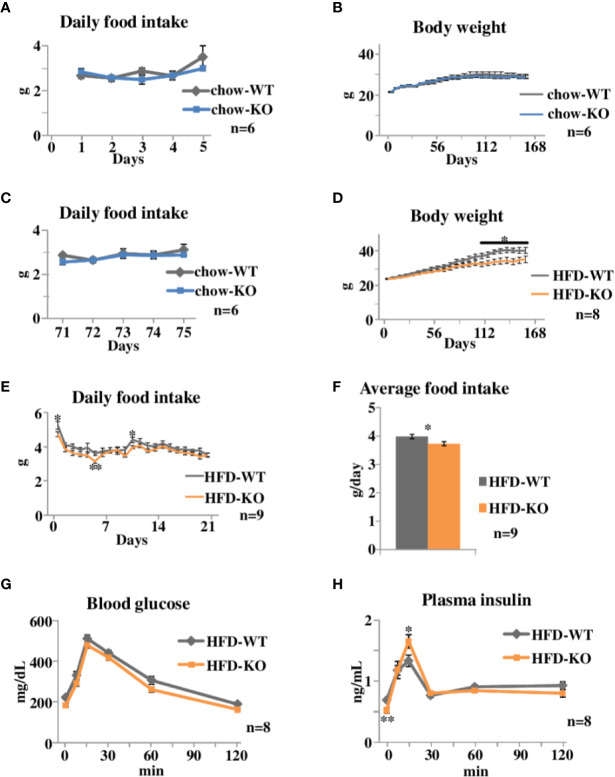
Time courses of body weight changes, food intake, and oral glucose tolerance tests in PAI-1 knockout (KO) mice fed chow or the safflower oil-rich high fat diet (HFD). **(A)** Daily food intake of chow-KO and chow-WT mice from the 1st day through the 5th day of observation. Each bar represents the mean ± SE of *n* = 6 mice. **(B)** Body weight of chow fed PAI-1 KO mice (chow-KO) and wild-type (WT) control mice (chow-WT). Eight-week-old mice were weighed weekly. Each bar represents the mean ± SE of *n* = 6 mice. **(C)** Daily food intake of chow-KO and chow-WT mice from the 71st day through the 75th day of observation. Each bar represents the mean ± SE of *n* = 6 mice. **(D)** Body weight of safflower oil-rich high fat diet (HFD) fed PAI-1 KO mice (HFD-KO) and WT control mice (HFD-WT). Eight-week-old mice were fed safflower oil-rich HFD and then were weighed weekly. Each bar represents the mean ± SE of *n* = 8 mice. **(E)** Daily food intake of HFD-KO and HFD-WT mice from the 1st day through the 21st day of observation. Each bar represents the mean ± SE of *n* = 9 mice. **(F)** Average food intake of HFD-KO and HFD-WT mice from the 1st day through the 21st day of observation. Each bar represents the mean ± SE of *n* = 9 mice. **(G)** Blood glucose levels and **(H)** plasma insulin levels during oral glucose tolerance tests performed 21 days after the initiation of safflower oil-rich HFD feeding. Results are expressed as mean ± SE of *n* = 8 mice. ^*^P < 0.05 and ^**^P < 0.01.

The inhibitory activity and specificity of TM5441 were previously described in detail (Boe et al., 2013). TM5441 was mixed with HFD powder, and then shaped into pellets. Given that mice eat approximately 4 g of HFD per day (**Figure 1E**), 108 mg of TM5441 per 900 g of HFD were mixed together when the mice were approximately 30 g each. This enabled the mice to consume approximately 15 mg of TM5441 per kg body weight per day. The TM5441 dosage was set at 15 mg/kg of body weight because TM5441 treatment at a dose of 20 mg/kg of body weight has been shown to suppress HFD-induced body weight gain without exerting adverse effects (Piao et al., 2016). Control mice were fed HFD pellets without TM5441.

### Experimental Procedures

#### Experimental Procedure 1

Eight-week-old PAI-1 KO mice (chow-KO mice) and wild type (WT) control mice (chow-WT mice) were fed the normal chow diet and were weighed weekly. Daily food intake was measured for 5 consecutive days from the 1st day and from the 71st day of observation.

#### Experimental Procedure 2

Eight-week-old PAI-1 KO mice (HFD-KO mice) and WT control mice (HFD-WT mice) were fed safflower oil-rich HFD and were weighed weekly. Daily food intake was measured for 21 consecutive days from the 1st day of observation. Oral glucose tolerance tests were performed on the 22nd day.

#### Experimental Procedure 3

Eight-week-old WT mice were fed safflower oil-rich HFD or lard-rich HFD and were weighed weekly.

#### Experimental Procedure 4

Eight-week-old PAI-1 KO mice were fed lard-rich HFD for 2 weeks. They were then divided into two groups. The first group was fed HFD mixed with TM5441 (HFD-KO-TM5441 mice), while the second group continued to eat HFD without TM5441 (HFD-KO-Control mice). Mice were weighed weekly and sacrificed on day 35 to remove the liver and WAT.

#### Experimental Procedures 5–10

Eight-week-old WT mice were fed lard-rich HFD for 2 weeks. They were then divided into two groups. The first group was fed HFD mixed with TM5441 (HFD-WT-TM5441 mice), while the second group continued to eat the HFD without TM5441 (HFD-WT-Control mice).

For experimental procedure 5, mice were weighed weekly. Blood was collected on day 7 to measure plasma leptin levels. Liver weight and WAT weight were measured on day 7 by using another set of mice.

For experimental procedure 6, spontaneous locomotor activities were measured on day 7.

For experimental procedure 7, oral glucose tolerance tests were performed on day 35.

For experimental procedure 8, all mice were given a daily intraperitoneal injection of either leptin (HFD-WT-TM5441-Leptin mice and HFD-WT-Control-Leptin mice) or saline (HFD-WT-TM5441-Saline mice and HFD-WT-Control-Saline mice) on day 7. The injections were continued once daily for 4 more days until day 11. Body weight was measured both before the injection on day 7 and 24 h after the injection on day 11.

For experimental procedure 9, all mice were fasted on day 7 for 24 h. On day 8, which was the day after the 24-h fast, mice were given a single injection of either leptin or saline. Four hours after this injection, the mice were sacrificed by decapitation to remove the brain.

For experimental procedure 10, all mice were sacrificed on day 7 to remove BAT.

### Blood Analysis

Blood samples, 30 µl per mouse, were collected by incision of the tail vein with a lance. Blood was collected into 0.5 ml Eppendorf tubes containing 0.2 μl of 0.5 M ethylenediaminetetraacetic acid (EDTA) and then centrifuged for 15 min at 8,000 rpm at 4°C to obtain plasma. Plasma leptin levels were determined with enzyme linked immunosorbent assay (ELISA) kits (Morinaga Institute of Biological Science, Yokohama, Japan) (Chiba et al., 2016). Plasma, 5 μl per mouse, was used for measuring leptin by ELISA. None of the samples required dilution for the assay. The detection range of the kit was 0.4–25.6 ng/ml.

### Oral Glucose Tolerance Tests

Blood glucose levels were assayed using a Glutestmint kit (Sanwa Kagaku Kenkyusho, Nagoya, Japan) (Uno et al., 2006). Plasma insulin levels were determined with ELISA kits (Morinaga Institute of Biological Science, Yokohama, Japan) (Uno et al., 2006). Plasma, 5 μl per mouse, was used for measuring insulin by ELISA. None of the samples required dilution for the assay. The detection range of the kit was 0.156–10 ng/ml. Mice that had been fasted for 9 h during daylight hours were given glucose at a dose of 2 g/kg body weight (Yamada et al., 2006). Blood glucose and plasma insulin were measured both immediately before administration and then again 15, 30, 60, 90, and 120 min after glucose loading.

### Locomotor Activity

Spontaneous locomotor activities of mice were analyzed with an infrared activity monitor (Supermex; Muromachi Kikai, Tokyo, Japan) as previously reported (Tsukita et al., 2012).

### Histological Analysis

Livers and white adipose tissues (WAT) were removed, fixed with 10% formalin and embedded in paraffin (Tsukita et al., 2012). Tissue sections were stained with hematoxylin eosin (HE). Adipocyte size was measured by the diameters of the adipocytes in light-microscopy images (20×) (n = 50 adipocytes per section, two sections per mouse) and analyzed using Keyence BZ-X710 Analyzer software. Experiments were carried out in a blinded manner by masking the slide number and then conducting the evaluation in a random order (Drareni et al., 2018).

### Leptin Loading Tests

Leptin loading tests were carried out as previously described (Tsukita et al., 2012) with slight modifications. To examine the effects of leptin treatment on body weight change, mice were given daily intraperitoneal administrations of leptin (5 mg/kg of body weight; R&D Systems, Minneapolis, MN, USA) or saline for 5 consecutive days. The leptin dosage was set at 5 mg/kg of body weight, based on a previous report (Kabra et al., 2016). To examine the effects of leptin treatment on hypothalamic neuropeptide expressions, 24-h fasted mice were given a single injection of either leptin (5 mg/kg of body weight) or saline, and were then sacrificed by decapitation 4 h after the injection. Brains were immediately frozen in isopentane with dry ice and stored at −80°C until RNA purification.

### Laser Microdissection

Coronal cryostat sections (80 μm) of the hypothalamic arcuate nucleus (ARC) were placed on PEN-coated slides (Leica Microsystems, Wetzlar, Germany) (Tsukita et al., 2012). Laser microdissection was carried out on a Leica AS LMD (Leica Microsystems, Wetzlar, Germany) (Asai et al., 2017). Immediately after microdissection, total RNA was purified using an RNeasy Micro Kit (Qiagen, Valencia, CA, USA) (Tsukita et al., 2017).

### RNA Purification and Quantitative Real-Time PCR (qRT-PCR)

Complementary DNA was synthesized from 1 μg of total RNA using a QuantiTect Reverse Transcription Kit (Qiagen) and was evaluated using a real-time PCR quantitative system (Light Cycler Quick System 350S; Roche Diagnostics, Mannheim, Germany) (Takahashi et al., 2013). mRNA expression levels were normalized against the levels of *glyceraldehyde 3-phosphate dehydrogenase* (*gapdh*) for the hypothalamic ARC and *beta-actin* for brown adipose tissue. The sequences of the forward and reverse primers used were as follows: *agouti-related peptide* (*agrp*), forward, 5′-CCAGAGTTCCCAGGTCTAAG-3′; *agrp*, reverse, 5′-CGGTTCTGTGGATCTAGCA-3′; *neuropeptide y* (*npy*), forward, 5′-GCTTGAAGACCCTTCCATTGGTG-3′; *npy*, reverse, 5′-GGCGGAGTCCAGCCTAGTGG-3′; *proopiomelanocortin* (*Pomc*), forward, 5′-TCACCACGGAGAGCAACCT-3′; *Pomc*, reverse, 5′-CAGCGGAAGTGACCCATGA-3′; g*apdh*, forward, 5′-TGAAGGTCGGTGTGAACG-3′; g*apdh*, reverse, 5′-CCATTCTCGGCCTTGACT-3′; *uncoupling protein 1* (*ucp1*), forward, 5′-TACCAAGCTGTGCGATGT-3′; *ucp1*, reverse, 5′-AAGCCCAATGATGTTCAGT-3′; *peroxisome proliferative activated receptor gamma coactivator 1 alpha* (*pgc-1a*), forward, 5′-ATACCGCAAAGAGCACGAGAAG-3′; *pgc-1a*, reverse, 5′-CTCAAGAGCAGCGAAAGCGTCACAG-3′; *type II iodothyronine deiodinase* (*dio2*), forward, 5′-ACTCGGTCATTCTGCTCAA-3′; *dio2*, reverse, 5′- AAGCCCAATGATGTTCAGT-3′; *cell death-inducing DNA fragmentation factor alpha subunit-like effector A* (*cidea*), forward, 5′-ACTTCCTCGGCTGTCTCAATGT-3′; *cidea*, reverse, 5′- TATCGCCCAGTACTCGGAGCAT-3′; *PR domain containing 16* (*prdm16*), forward, 5′-TGTACAGGCAGGCTAAGAA-3′; *prdm16*, reverse, 5′-CCTGTGATGTTCAAGGAA-3′; *beta-actin*, forward, 5′-GATGCCCTGAGGCTCTT-3′; and *beta-actin*, reverse, 5′-TGTGTTGGCATAGAGGTCTTTAC-3′.

### Statistical Analysis

All values are expressed as mean ± SE. Data were compared employing Student’s t test to examine the effect of one factor, two-way ANOVA followed by Tukey’s test to examine the effects of two factors, or two-way repeat measures ANOVA to determine variance with respect to time and mouse group. The level of significance was set at *P* < 0.05.

## Results

### PAI-1 Deficiency Alleviates HFD-Induced Body Weight Gain

We first examined the effects of PAI-1 deficiency on body weight and food intake under normal chow feeding conditions (Experimental procedure 1). Eight-week-old PAI-1 KO mice (chow-KO mice) and wild type (WT) control mice (chow-WT mice) were fed the normal chow diet. Daily food intake from the 1st day (**Figure 1A**) of observation was similar in these two groups. Body weight of the two groups were similar throughout the observation period (**Figure 1B**), as expected from a previous report (Ma et al., 2004). Daily food intake from the 71st day (**Figure 1C**) of observation was also similar in these two groups. Therefore, PAI-1 deficiency itself appears to have almost no effects on either eating habits or body weight alterations under normal chow feeding conditions.

We next examined the effects of PAI-1 deficiency on body weight and food intake under HFD-fed conditions. Safflower oil-rich HFD was given to 8-week-old PAI-1 KO mice (HFD-KO mice) and WT mice (HFD-WT mice) (Experimental procedure 2). The HFD-KO mice gained less weight than the HFD-WT mice (Two-way repeat measures ANOVA; F = 4.53, *P* < 0.001, HFD-WT mice 40.3 ± 1.68 g *vs* HFD-KO mice 34.6 ± 1.84 g, *P* = 0.039), and the difference between the two groups became statistically significant after 106 days (**Figure 1D**). Strikingly, HFD-KO mice ate significantly less than HFD-WT mice before their body weights showed significant differences (Two-way repeat measures ANOVA; F = 35.89, *P* < 0.001) (**Figure 1E**) and the average food intake amounts during the experimental period (21 days) were also significantly smaller in HFD-KO mice than in HFD-WT mice (HFD-WT mice 3.98 ± 0.08 g/day *vs* HFD-KO mice 3.73 ± 0.07 g/day, *P* = 0.021) (**Figure 1F**). Since reduced food intake preceded the evident differences in body weight, reduced energy intake is likely to be a direct cause, rather than a result, of suppressed weight gain. Thus, PAI-1 deficiency may suppress HFD-induced weight gain, while exerting minimal effects on weight regulation under normal chow-fed conditions.

### PAI-1 Deficiency Improves Insulin Sensitivities and Insulin Secretion

We next performed oral glucose tolerance tests on HFD-KO mice and HFD-WT mice after 22 days of HFD feeding. Blood glucose levels of HFD-KO mice tended to be decreased as compared to those of HFD-WT mice (**Figure 1G**). Fasting plasma insulin levels were significantly lower in HFD-KO mice despite fasting blood glucose levels being similar (Two-way repeat measures ANOVA; F = 5.17, *P* = 0.036, HFD-WT mice 0.69 ± 0.03 ng/ml *vs* HFD-KO mice 0.52 ± 0.04 ng/ml, *P* = 0.005) (**Figure 1H**), indicating that these mice had better insulin sensitivity. In addition, plasma insulin levels 15 min after glucose loading were significantly higher in HFD-KO mice than in HFD-WT mice (HFD-WT mice 1.33 ± 0.09 ng/ml *vs* HFD-KO mice 1.65 ± 0.11 ng/ml, *P* = 0.048). Taken together, these results suggest that suppression of body weight gain in PAI-1 KO mice improves both insulin sensitivity and glucose-stimulated secretion of insulin.

### PAI-1 Inhibitor Treatment Alleviates HFD-Induced Body Weight Gain and Insulin Resistance

**Figure 2:**
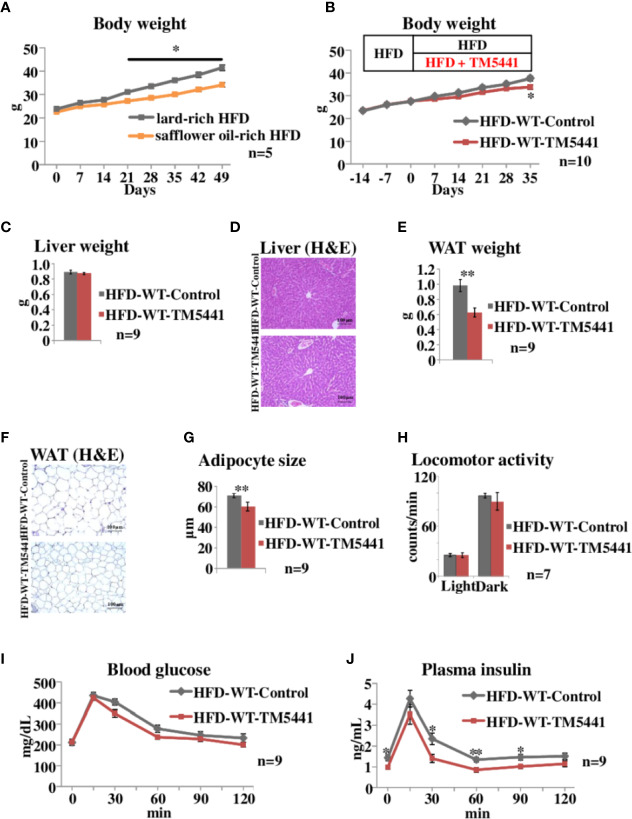
Body weight and oral glucose tolerance tests in lard-rich HFD fed mice treated with TM5441. **(A)** Body weight of WT mice fed safflower oil-rich HFD or lard-rich HFD. Each bar represents the mean ± SE of *n* = 5 mice. **(B)** Body weight of lard-rich HFD-fed mice treated with TM5441. Ten-week-old WT mice that had been pre-fed the lard-rich HFD for 2 weeks were given either lard-rich HFD mixed with TM5441 (HFD-WT-TM5441 mice) or lard-rich HFD (HFD-WT-Control mice) and then were weighed weekly. Each bar represents the mean ± SE of *n* = 10 mice. **(C)** Liver weight of HFD-WT-TM5441 and HFD-WT-Control mice. Each bar represents the mean ± SE of *n* = 9 mice. **(D)** Representative hematoxylin eosin (HE) staining of liver sections of HFD-WT-TM5441 and HFD-WT-Control mice (original magnification: 20×, scale bars: 100 μm). **(E)** Epididymal white adipose tissue (WAT) weight of HFD-WT-TM5441 and HFD-WT-Control mice. Each bar represents the mean ± SE of *n* = 9 mice. **(F)** Representative HE staining of WAT sections of HFD-WT-TM5441 and HFD-WT-Control mice (original magnification: 20×, scale bars: 100 μm). **(G)** Adipocyte size of HFD-WT-TM5441 and HFD-WT-Control mice. Each bar represents the mean ± SE of *n* = 9 mice. **(H)** Locomotor activities of HFD-WT-TM5441 and HFD-WT-Control mice during light hours and dark hours. Each bar represents the mean ± SE of *n* = 7 mice. **(I)** Blood glucose levels and **(J)** plasma insulin levels during oral glucose tolerance tests performed 35 days after the initiation of lard-rich HFD feeding. Results are expressed as mean ± SE of *n* = 9 mice. ^*^P < 0.05 and ^**^P < 0.01.

We then examined the effects of TM5441, a selective PAI-1 inhibitor, on body weight in HFD-fed mice. In the following experiments, we switched from safflower oil-rich HFD to lard-rich HFD (Experimental procedure 3), because mice fed the latter gained more weight than those given the former (Two-way repeat measures ANOVA; F = 12.38, *P* < 0.001, lard-rich HFD mice 41.5 ± 1.21 g *vs* safflower oil-rich HFD mice 34.1 ± 0.98 g on day 49, *P* = 0.003) (**Figure 2A**). Ten-week-old WT mice that had been pre-fed a lard-rich HFD for 2 weeks were divided into two groups. The first group was then fed HFD mixed with TM5441 (HFD-WT-TM5441 mice), while the second group continued to eat the HFD without TM5441 (HFD-WT-Control mice) (Experimental procedure 5). No death, hair loss, signs of respiratory infections such as open-mouth breathing, or other adverse effects were observed in any of the TM5441 treated mice. Body weight gain was slower in mice that ate HFD mixed with TM5441, and these reductions reached statistical significance 35 days after the initiation of inhibitor treatment (Two-way repeat measures ANOVA; F = 5.24, *P* < 0.001, HFD-WT-Control mice 37.6 ± 1.07 g *vs* HFD-WT-TM5441 mice 33.8 ± 0.97 g on day 35, *P* = 0.017) (**Figure 2B**). Liver weights on day 7 were similar in the two groups (**Figure 2C**), and there was no evidence of either hepatocyte ballooning or lobular inflammation in the two groups (**Figure 2D**). On the other hand, epididymal white adipose tissue (WAT) weight on day 7 was significantly lower in HFD-WT-TM5441 mice than in HFD-WT-Control mice (HFD-WT-Control mice 0.98 ± 0.08 g *vs* HFD-WT-TM5441 mice 0.63 ± 0.06 g, *P* = 0.003) (**Figure 2E**). On average, adipocytes were markedly smaller in HFD-WT-TM5441 mice (HFD-WT-Control mice 71.05 ± 1.49 μm *vs* HFD-WT-TM5441 mice 60.35 ± 2.89 μm, *P* = 0.003) (**Figures 2F, G**), indicating decreased accumulation of lipid per adipocyte in HFD-WT-TM5441 mice. Thus, TM5441 treatment alleviates the weight gain induced by HFD, after reducing WAT weight. If loss of lean muscle mass had occurred, it might also have contributed to reduced body weight gain. Although direct measurement of lean muscle mass will be required to examine this possibility, spontaneous locomotor activities on day 7, during both light hours and dark hours, were similar in these groups of mice (light hours: HFD-WT-Control mice 25.68 ± 1.81 counts/min *vs* HFD-WT-TM5441 mice 25.56 ± 2.76 counts/min, *P* = 0.972, dark hours: HFD-WT-Control mice 96.88 ± 2.93 counts/min *vs* HFD-WT-TM5441 mice 90.06 ± 10.53 counts/min, *P* = 0.544) (**Figure 2H**) (Experimental procedure 6), which might suggest that severe loss of muscle mass would have been unlikely to have occurred in HFD-WT-TM5441 mice.

Next, HFD-WT-TM5441 mice and HFD-WT-Control mice were subjected to oral glucose tolerance tests on day 35 (Experimental procedure 7). Blood glucose levels of HFD-WT-TM5441 mice tended to be lower than those of HFD-WT-Control mice (**Figure 2I**). Plasma insulin levels were significantly decreased in HFD-WT-TM5441 mice as compared to HFD-WT-Control mice (Two-way repeat measures ANOVA; F = 5.17, *P* = 0.036, 0 min: 1.43 ± 0.14 ng/ml *vs* 0.98 ± 0.09 ng/ml, *P* = 0.017, 30 min: 2.35 ± 0.28 ng/ml *vs* 1.40 ± 0.19 ng/ml, *P* = 0.015, 60 min: 1.34 ± 0.12 ng/ml *vs* 0.85 ± 0.11 ng/ml, *P* = 0.007, 90 min: 1.47 ± 0.14 ng/ml *vs* 1.01 ± 0.10 ng/ml, *P* = 0.021, HFD-WT-Control mice *vs* HFD-WT-TM5441 mice, respectively) (**Figure 2J**). These results suggest that insulin sensitivity of HFD-WT-TM5441 mice is increased as compared to that of HFD-WT-Control mice.

Taken together, these results indicate that pharmacological inhibition of PAI-1 activity after initiation of HFD feeding attenuates body weight gain and improves insulin sensitivity.

### HFD-Induced Body Weight Gain Is Attenuated by TM5441 Treatment Through a PAI-1 Dependent-Mechanism

**Figure 3:**
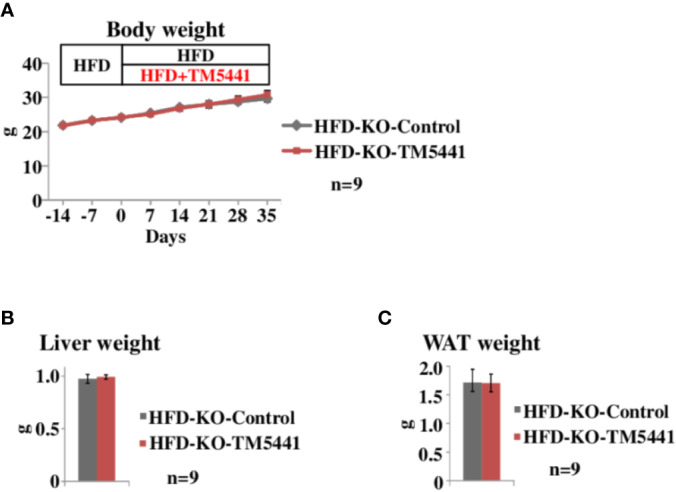
Body weight of lard-rich HFD fed PAI-1 knockout (KO) mice treated with TM5441. **(A)** Body weight of lard-rich HFD-fed PAI-1 KO mice treated with TM5441. Ten-week-old PAI-1 KO mice that had been pre-fed the lard-rich HFD for 2 weeks were given either lard-rich HFD mixed with TM5441 (HFD-KO-TM5441 mice) or lard-rich HFD (HFD-KO-Control mice) and then were weighed weekly. Each bar represents the mean ± SE of *n* = 9 mice. **(B)** Liver weight of HFD-KO-TM5441 and HFD-KO-Control mice. Each bar represents the mean ± SE of *n* = 9 mice. **(C)** WAT weight of HFD-KO-TM5441 and HFD-KO-Control mice. Each bar represents the mean ± SE of *n* = 9 mice.

To rule out the possibility that body weight reduction seen in HFD-WT-TM5441 mice (**Figure 2B**) was a consequence of non-specific effects of TM5441, i.e. effects other than PAI-1 inhibition, such as an unpleasant taste, we treated HFD-fed PAI-1 KO mice with TM5441 (Experimental procedure 4). Ten-week-old PAI-1 KO mice that had been pre-fed HFD for 2 weeks were divided into two groups. The first group was then fed HFD mixed with TM5441 (HFD-KO-TM5441 mice), while the second group continued to eat HFD without TM5441 (HFD-KO-Control mice). As expected, weekly body weights of HFD-KO-TM5441 mice after TM5441 loading were almost equal to those in HFD-KO-Control mice (**Figure 3A**). In addition, not only liver but also epididymal WAT weights on day 35 were similar in the two groups (**Figures 3B, C**). Thus, anti-obesity effects of TM5441 treatment in HFD-fed mice were observed only in the presence of intrinsic PAI-1 (**Figure 2B**). Thus, TM5441 was confirmed to exert its anti-obesity effects *via* inhibition of PAI-1 activity. In addition, these findings clearly indicate that treatment with a PAI-1 inhibitor after obesity development is also effective for suppressing weight gain in mice that are not depleted of PAI-1.

### PAI-1 Inhibitor Treatment Improves Leptin Sensitivity

**Figure 4:**
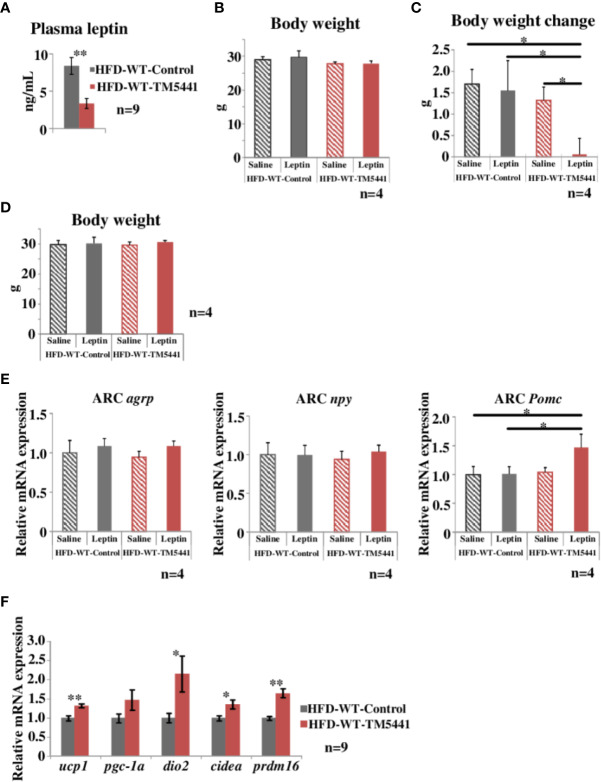
Evaluation of leptin sensitivity in lard-rich HFD fed mice treated with TM5441. **(A)** Plasma leptin levels of HFD-WT-TM5441 mice and HFD-WT-Control mice. Results are expressed as mean ± SE of *n* = 9 mice. **(B)** Body weight of HFD-WT-TM5441-Leptin mice, HFD-WT-Control-Leptin mice, HFD-WT-TM5441-Saline mice, and HFD-WT-Control-Saline mice. Ten-week-old WT mice that had been pre-fed the lard-rich HFD for 2 weeks were given either lard-rich HFD mixed with TM5441 or lard-rich HFD for 1 week. Results are expressed as mean ± SE of *n* = 4 mice. **(C)** Body weight changes during leptin treatment. Mice were given a daily intraperitoneal injection of either leptin (HFD-WT-TM5441-Leptin mice or HFD-WT-Control-Leptin mice) or saline (HFD-WT-TM5441-Saline mice or HFD-WT-Control-Saline mice) for 5 consecutive days. Body weight immediately before the first leptin injection was subtracted from body weight 24 h after the last injection. Results are expressed as mean ± SE of *n* = 4 mice. **(D)** Body weight of HFD-WT-TM5441-Leptin mice, HFD-WT-Control-Leptin mice, HFD-WT-TM5441-Saline mice, and HFD-WT-Control-Saline mice. Ten-week-old WT mice that had been pre-fed the lard-rich HFD for 2 weeks were given either lard-rich HFD mixed with TM5441 or lard-rich HFD for 1 week. Results are expressed as mean ± SE of *n* = 4 mice. **(E)** Relative mRNA expressions of *agouti-related peptide* (*agrp*), *neuropeptide y* (*npy*), and *proopiomelanocortin* (*Pomc*) in the hypothalamic arcuate nucleus (ARC). Mice were fasted for 24 h and then given a single intraperitoneal injection of either leptin (HFD-WT-TM5441-Leptin mice or HFD-WT-Control-Leptin mice) or saline (HFD-WT-TM5441-Saline mice or HFD-WT-Control-Saline mice). Brain samples were harvested 4 h after the injection. Results are expressed as mean ± SE of *n* = 4 mice. **(F)** Relative mRNA expressions of *uncoupling protein 1* (*ucp1*), *peroxisome proliferative activated receptor gamma coactivator 1 alpha* (*pgc-1a*), *type II iodothyronine deiodinase* (*dio2*), *cell death-inducing DNA fragmentation factor alpha subunit-like effector A* (*cidea*), and *PR domain containing 16* (*prdm16*) in brown adipose tissue (BAT). Ten-week-old WT mice that had been pre-fed the lard-rich HFD for 2 weeks were given either lard-rich HFD mixed with TM5441 or lard-rich HFD for 1 week. Results are expressed as mean ± SE of *n* = 9 mice. ^*^P < 0.05 and ^**^P < 0.01.

Leptin functions as an afferent signal in a negative feedback loop of energy metabolism leading to reduced feeding and promotion of energy expenditure (Friedman, 2016). However, hypothalamic leptin sensitivity is known to be impaired despite elevated circulating leptin concentrations under conditions of obesity (Cui et al., 2017), a phenomenon regarded as leptin resistance. Leptin resistance is considered to further promote body weight gain through a feed-forward system (Yamada et al., 2013). Hence, we measured circulating levels of leptin in HFD-WT-TM5441 mice and HFD-WT-Control mice 1 week after TM5441 loading (Experimental procedure 5). At this time-point, although body weights were similar in the two groups (day 7 of **Figure 2B**), interestingly, plasma leptin levels in HFD-WT-TM5441 mice had already decreased to less than half of those in HFD-WT-Control mice (HFD-WT-Control mice 8.38 ± 1.13 ng/ml *vs* HFD-WT-TM5441 mice 3.37 ± 0.68 ng/ml, *P* = 0.002) (**Figure 4A**). In addition, during the following weeks, HFD-WT-TM5441 mice gained less body weight than HFD-WT-Control mice. Therefore, we hypothesized that TM5441 treatment alleviated leptin resistance under HFD-fed conditions. To examine this possibility, we administered exogenous leptin to these mice, followed by monitoring body weights (Experimental procedure 8). After 1 week of HFD with or without TM5441, all mice were given a daily intraperitoneal injection of either leptin (HFD-WT-TM5441-Leptin mice and HFD-WT-Control-Leptin mice) or saline (HFD-WT-TM5441-Saline mice and HFD-WT-Control-Saline mice) for 5 consecutive days. There were no differences in body weights between the mice group before the first day of leptin or saline injection (**Figure 4B**). Under these settings, no differences in body weight change after leptin or saline injection were seen between HFD-WT-Control-Saline mice and HFD-WT-Control-Leptin mice (**Figure 4C**), suggesting that these HFD-fed mice have severe leptin resistance. Interestingly, administration of leptin after TM5441 treatment resulted in a remarkable suppression of body weight gain; HFD-WT-TM5441-Leptin mice gained markedly less weight than any of the other groups (HFD-WT-Control-Saline mice 1.70 ± 0.34 g *vs* HFD-WT-TM5441-Leptin mice 0.06 ± 0.38 g, *P* = 0.013, HFD-WT-Control-Leptin mice 1.55 ± 0.70 g *vs* HFD-WT-TM5441-Leptin mice 0.06 ± 0.38 g, *P* = 0.030, HFD-WT-TM5441-Saline mice 1.32 ± 0.31 g *vs* HFD-WT-TM5441-Leptin mice 0.06 ± 0.38 g, *P* = 0.049) (**Figure 4C**). Because body weights of all four groups of mice were similar on the first day of leptin or saline injection (**Figure 4B**), improvement of leptin sensitivity is suggested to be a direct effect of PAI-1 inhibition rather than a secondary effect reflecting suppressed weight gain. Thus, TM5441 treatment primarily improves leptin sensitivity in HFD-fed mice, thereby suppressing weight gain.

We next investigated the effect of TM5441 on leptin receptor signaling in the hypothalamus in HFD-fed mice (Experimental procedure 9). Leptin binds to the leptin receptor on proopiomelanocortin (POMC) neurons in the arcuate nucleus (ARC) to directly activate and induce POMC expression, while exerting an inhibitory effect on agouti-related peptide (AgRP)/neuropeptide Y (NPY) neurons (Timper and Bruning, 2017). We therefore studied ARC of HFD-WT-TM5441-Leptin, HFD-WT-Control-Leptin, HFD-WT-TM5441-Saline, and HFD-WT-Control-Saline mice fed HFD for 1 week, which had similar body weight (**Figure 4D**). Expression levels of these neuropeptides were examined in the ARC of mice 4 h after leptin administration. Although mRNA expression levels of *agrp* and *npy* showed no alterations in these four groups, *Pomc* expression was significantly increased in HFD-WT-TM5441-Leptin mice as compared to WT-Control-Leptin mice (HFD-WT-Control-Leptin mice 1.01 ± 0.12 *vs* HFD-WT-TM5441-Leptin mice 1.47 ± 0.23, *P* = 0.031) (**Figure 4E**). We further studied the effect of TM5441 on expressions of thermogenesis-related genes, such as *ucp1*, *dio2*, *cidea*, and *prdm16*, in brown adipose tissue (BAT) (Experimental procedure 10), because enhanced leptin signaling in the hypothalamic ARC increases BAT thermogenesis (Rezai-Zadeh and Munzberg, 2013). After 1 week of HFD with or without TM5441, all of the thermogenesis-related genes, including *ucp 1* (HFD-WT-Control mice 1.00 ± 0.07 *vs* HFD-WT-TM5441 mice 1.32 ± 0.05, *P* = 0.002), were significantly up-regulated by TM5441 treatment (**Figure 4F**). These findings, taken together, suggest that administering TM5441 improves leptin sensitivity in neurons comprising the hypothalamic ARC, thereby activating BAT. Notably, this improvement was also observed, before suppression of weight gain became evident. Thus, an underlying mechanism whereby TM5441 treatment suppresses weight gain is alleviation of hypothalamic leptin resistance induced by HFD.

## Discussion

In the present study, we identified a previously unknown role of PAI-1 in body weight regulation. First, under HFD fed conditions, PAI-1 KO mice gained less body weight, and significant reduction in food intake preceded weight gain. We next found that pharmacological inhibition of PAI-1 during HFD feeding also suppressed HFD-induced body weight gain. TM5441 did not suppress HFD-induced body weight gain in PAI-1-KO mice, indicating that PAI-1 inhibition *per se* is the mechanism underlying suppression of HFD-induced body weight gain. Finally, we found that leptin sensitivity was improved by TM5441 treatment. Therefore, the mechanism underlying weight gain suppression by PAI-1 inhibition involves alleviation of leptin resistance. Collectively, these observations indicate that PAI-1 plays a role in aggravating leptin resistance during obesity development. Therefore, PAI-1 inhibitors may act as leptin sensitizers in states of HFD-induced obesity. Thus, inhibition of PAI-1 activity is a potential therapeutic strategy for fighting obesity *via* alleviation of leptin resistance.

Contrary to HFD fed conditions, neither weight gain nor food intake was reduced in PAI-1 KO mice under chow fed conditions. These features might be explained by a difference in PAI-1 expressions between lean and obese states. PAI-1 is produced in adipose tissue (Khandekar et al., 2011) and its PAI-1 expression is increased in states of obesity, since several fold higher *pai-1* expressions are seen in adipose tissues of HFD fed mice than in those of chow fed mice (Wang et al., 2018). Consistently, elevated PAI-1 levels are seen in both adipose tissues and plasma obtained from obese mice and humans as compared to lean controls (Bodary, 2007). Therefore, marked effects of PAI-1 deficiency or PAI-1 inhibition might not be observable under chow fed conditions when PAI-1 is not elevated.

In addition to suppression of body weight gain, PAI-1 deletion and PAI-1 inhibition resulted in improvement of insulin sensitivity. Because improved insulin sensitivity secondary to weight loss is a significant contributor to the improvement and reversal of type 2 diabetes mellitus (T2DM) (Chaudhry et al., 2016), our findings suggest that PAI-1 might be involved in the pathophysiology of T2DM and insulin resistance.

HFD-induced leptin resistance is associated with chronic low-grade inflammation in the hypothalamus (Saltiel, 2016). Although it remains unclear whether PAI-1 passes through the blood brain barrier, elevated PAI-1 in the circulation may exacerbate the hypothalamic inflammation during obesity development. In addition to this mechanism, PAI-1 which is produced within the brain by microglial cells may underlie the enhancement of hypothalamic inflammation (Jeon et al., 2012). Increased saturated fatty acids in the periphery reportedly cross the blood brain barrier and induce inflammatory responses in hypothalamic neurons, as rapidly as within a few days after HFD initiation and obviously before substantial weight gain occurs (Thaler et al., 2012). Thus, fatty acids are considered to be involved in inducing hypothalamic inflammation. Inflammatory processes in the hypothalamus reportedly involves activation of microglia, the tissue-resident macrophages in the brain, which further secrete inflammatory cytokines, such as tumor necrosis factor-α, in the hypothalamus (Avalos et al., 2018). PAI-1 might be another cytokine that contributes to hypothalamic inflammation, because resident cells in the brain including microglia reportedly produce PAI-1 (Arnoldussen et al., 2014). In addition, microglial migration is considered to be enhanced by autocrine or paracrine of PAI-1, because (i) PAI-1 treatment promoted migration of microglial cell lines *in vitro* in a dose-dependent manner, (ii) PAI-1 exerted these effects *in vitro* by targeting low density lipoprotein receptor-related protein-1 in microglial cell lines, and (iii) exogenous PAI-1 injection actually promoted microglial migration *in vivo* (Jeon et al., 2012). Thus, HFD feeding might initiate hypothalamic inflammation, and then induce activation and migration of microglia, which in turn results in autocrine or paracrine secretion of PAI-1, leading to further enhancement of microglial migration and hypothalamic inflammation, thereby forming a vicious cycle. Importantly, TM5441 has already been shown to penetrate the blood brain barrier and thereby reach the central nervous system (Pelisch et al., 2015). This mechanism may explain why PAI-1 deletion had no effect in chow-fed mice (**Figures 1A–C**), while both PAI-1 deletion (**Figures 1D, F**) and pharmacological inhibition (**Figures 2A, B**) exerted profound effects in HFD-fed mice. At present, we do not have experimental data supporting the presence of chronic systemic inflammation or hypothalamic local inflammation in HFD-fed control mice. Future studies focusing on HFD-induced hypothalamic inflammation, especially whether such inflammatory responses are affected by PAI-deletion or TM5441 treatment, might more precisely unveil molecular mechanism by which PAI-1 modulates HFD-induced leptin resistance.

Leptin decreases body weight by inhibiting food intake as well as by promoting energy expenditure by acting on hypothalamic nuclei (Timper and Bruning, 2017). Among these nuclei, the ARC is the key area for the regulation of feeding behavior (Saltiel, 2016). In this study, decreased plasma leptin concentrations were observed in HFD-WT-TM5411 mice 7 days after TM 5411 treatment (**Figure 4A**), while the body weight of HFD-WT-TM5411 mice was not changed at this time-point (day 7 of **Figure 2B**). If leptin sensitivity is not changed by TM5441 treatment, decreased leptin levels in these mice would theoretically induce further body weight gain. However, no induction of body weight gain was observed in HFD-WT-TM5411 mice. Instead, weight gain was actually suppressed. These findings suggest that improvement of leptin sensitivity primarily occurred in HFD-WT-TM5411 mice and led to attenuation of their body weight gain. Expectedly, leptin tolerance tests, which had been performed before body weight differences became evident, directly demonstrated leptin sensitivity to be markedly improved by TM5411 treatment (**Figures 4B, C**). TM5411 treatment significantly increased POMC expressions in ARC after leptin loading. In addition, genetic deletion of PAI-1 suppressed food intake before body weight differences became evident under HFD-fed conditions. These findings, taken together, suggest that inhibition of PAI-1 activity attenuates hypothalamic leptin resistance induced by HFD feeding, thereby inducing body weight reduction. This mechanism, alleviation of leptin resistance, might be ideal for suppressing weight gain without exerting adverse effects, such as hypophagia once the individual has returned to a lean state and leptin resistance has disappeared. This concept is likely supported by results indicating that even total PAI-1 deficiency did not affect energy metabolism in chow-fed mice that were non-obese and leptin-sensitive (**Figures 1A–C**).

Seven drugs are currently approved by the U.S. Food and Drug Administration for treating obesity (Saltiel, 2016). However, all of these drugs were reported to have adverse effects limiting their usefulness, particularly because most reduced caloric intake by targeting neurons that control not only appetite but also sleep and wakefulness, drug dependence, and other reward systems (Saltiel, 2016). None of these drugs directly target the leptin sensing systems nor do they primarily alleviate leptin resistance. Strategies for overcoming leptin resistance are very limited even in animal models; only those that have focused on (i) development of protein tyrosine phosphatase inhibitors to increase the leptin-induced signal transducer and activator of transcription 3 signaling (Loh et al., 2011) or (ii) targeting of melanocortin pathways, a downstream target of POMC neurons (Pierroz et al., 2002), have been proposed. Therefore, pharmacological inhibition of PAI-1 activity might emerge as a novel, promising and safe strategy for suppressing body weight gain, if clinical trials demonstrate TM5441 to be both effective and safe in humans.

## Conclusion

We have demonstrated a novel role of PAI-1 in exacerbating hypothalamic leptin resistance, resulting in HFD-induced obesity. Furthermore, we discovered a novel beneficial effect of PAI-1 inhibitor treatment, i.e. improved leptin sensitivity, in mice with HFD-induced obesity. Drugs with this mechanism of action might revolutionize therapeutic approaches to obesity worldwide.

## Data Availability Statement

The raw data supporting the conclusions of this article will be made available by the authors, without undue reservation.

## Ethics Statement

The animal study was reviewed and approved by Institutional Animal Care and Use Committee of the Tohoku University Environmental & Safety Committee.

## Author Contributions

SH, TY, and KT conducted the research and obtained the data, contributed to relevant discussions, wrote the manuscript, and reviewed/edited the manuscript. TD and TM synthesized TM5411 and contributed to the relevant discussions. KK, SK, YA, YM, AE HS, YK, JY, TI, SS, and JI contributed to the relevant discussions. HK supervised the research, contributed to the relevant discussions, wrote the manuscript, and reviewed/edited the manuscript.

## Funding

This work was supported in part by Grant-in-Aid for Scientific Research on Innovative Areas (Thermal Biology) (15H05932) to TY from MEXT, Grants-in-Aid for Scientific Research to HK (17H01565), TY (18H02859 and 26293215), and KT (19K17951 and 16K19529) from the Japan Society for the Promotion of Science of Japan. This research was also supported by the Japan Agency for Medical Research and Development, AMED, under Grant Number JP19gm5010002.

## Conflict of Interest

The authors declare that the research was conducted in the absence of any commercial or financial relationships that could be construed as a potential conflict of interest.
